# Cross-Reactive Fc-Fused Single-Domain Antibodies to Hemagglutinin Stem Region Protect Mice from Group 1 Influenza a Virus Infection

**DOI:** 10.3390/v14112485

**Published:** 2022-11-10

**Authors:** Daria V. Voronina, Dmitry V. Shcheblyakov, Irina A. Favorskaya, Ilias B. Esmagambetov, Alina S. Dzharullaeva, Amir I. Tukhvatulin, Olga V. Zubkova, Olga Popova, Vladislav Y. Kan, Alina S. Bandelyuk, Maxim M. Shmarov, Denis Y. Logunov, Boris S. Naroditskiy, Aleksandr L. Gintsburg

**Affiliations:** 1 Department of Genetics and Molecular Biology of Bacteria, National Research Center for Epidemiology and Microbiology Named after the Honorary Academician N. F. Gamaleya, 123098 Moscow, Russia; 2 Medical Microbiology Department, National Research Center for Epidemiology and Microbiology Named after the Honorary Academician N. F. Gamaleya, 123098 Moscow, Russia

**Keywords:** influenza, single-domain antibody, VHH, Fc-fusion

## Abstract

The continued evolution of influenza viruses reduces the effectiveness of vaccination and antiviral drugs. The identification of novel and universal agents for influenza prophylaxis and treatment is an urgent need. We have previously described two potent single-domain antibodies (VHH), G2.3 and H1.2, which bind to the stem domain of hemagglutinin and efficiently neutralize H1N1 and H5N2 influenza viruses in vivo. In this study, we modified these VHHs with Fc-fragment to enhance their antiviral activity. Reformatting of G2.3 into bivalent Fc-fusion molecule increased its in vitro neutralizing activity against H1N1 and H2N3 viruses up to 80-fold and, moreover, resulted in obtaining the ability to neutralize H5N2 and H9N2 subtypes. We demonstrated that a dose as low as 0.6 mg/kg of G2.3-Fc or H1.2-Fc administered systemically or locally before infection could protect mice from lethal challenges with both H1N1 and H5N2 viruses. Furthermore, G2.3-Fc reduced the lung viral load to an undetectable level. Both VHH-Fc antibodies showed in vivo therapeutic efficacy when delivered via systemic or local route. The findings support G2.3-Fc as a potential therapeutic agent for both prophylaxis and therapy of Group 1 influenza A infection.

## 1. Introduction

Influenza remains one of the major burdens in global healthcare, leading to 3–5 million severe illnesses and up to 650,000 lethal cases annually [1]. Annual seasonal epidemics are commonly caused by influenza virus types A and B, with a predominance of influenza A viruses (IAV). The high prevalence among the human population and the ability to cause pandemics that take millions of human lives make IAV of high epidemiological importance. The repeated seasonal epidemics and the chance of unpredictable pandemic outbreaks are associated with the antigenic evolution of IAV, which facilitates the escape from pre-existing immunity induced by prior infection or vaccination, and therefore reduces the effectiveness of vaccination [2,3,4]. The high rate of mutation due to the RNA nature of the influenza virus genome results in antiviral drug resistance [5,6,7,8]. Thus, there is an urgent need to develop novel and effective antiviral agents for the treatment and prophylaxis of IAV infection.

Hemagglutinin (HA) is a surface glycoprotein, which ensures attachment and viral entry into the host cell, and is one of the primary targets for immune response. Phylogenetically, HA molecules can be classified into two groups: Group 1 (H1, H2, H5, H6, H8, H9, H11, H12, H13, H16, H17, and H18) and Group 2 (H3, H4, H7, H10, H14, and H15) [9]. HA consists of two structurally and functionally distinct domains, variable globular head, and conserved stem domain. The immunodominant globular head region is very susceptible to accumulating mutations under the pressure of an antibody response [10,11,12,13,14,15], and many of these antibodies are strain-specific. However, antibodies directed against the HA stem can bind a broad range of influenza subtypes, but are not usually produced in high titers [16,17,18]. Therefore, the НА stem domain is an attractive target for both specific prophylaxis and therapy, with the potency to obtain an agent with broad-spectrum specificity.

Monoclonal antibodies (mAbs) have been proven to be effective therapeutics for both non-infectious and infectious diseases [19,20,21,22]. Among different types of antibodies, camelid- or shark-derived single-domain antibodies (sdAbs) have several advantages over conventional mAbs in the treatment of respiratory infections, including influenza virus infection. SdAbs represent variable fragments of heavy-chain antibody, which recognize the antigen with only one domain, named VHH. Previous studies have reported the development of promising therapeutic VHH to various influenza antigens [23,24,25,26,27], in particular, broadly reactive anti-HA stem VHHs [28,29,30,31]. Due to their unique CDR3 region forming an extended loop, VHHs have superiority over conventional immunoglobulins in binding hidden epitopes that may not be accessible for mAbs [32,33]. Moreover, sdAbs possess a low immunogenicity risk profile, making them promising for future potential therapeutic applications [34].

We have previously described two anti-HA stem VHHs—G2.3 and H1.2—obtained by immunization of alpacas with the human influenza seasonal vaccine, which are efficient in in vivo neutralization of H1N1 and H5N2 influenza subtypes [35]. However, the small size of monomeric VHHs results in a short half-life of approximately 90 min in vivo [36], which limits their therapeutic or prophylactic applications [37]. Several strategies have been described for modification of VHHs to improve their pharmacokinetics [36], and one of the frequently used approaches is a fusion of sdAb to an antibody Fc region [38]. An important role in limiting the development of influenza infection is played by the effector functions of antibodies mediated by the Fc-fragment, antibody-dependent cellular cytotoxicity (ADCC) and antibody-dependent cellular phagocytosis (ADCP) [39,40,41,42,43,44,45]. Hence, VHH is a promising tool for influenza therapy, but it is of great importance to optimize the structure of sdAbs in order to increase their half-life and obtain effector functions by modification with an Fc-fragment.

Most influenza therapeutic mAbs are administered via systemic routes, typically intravenously, and in the case of animal studies, intraperitoneal (i.p.) administration is the predominant route. However, the main targets for influenza virus and the main affected area are the upper and lower respiratory tracts [46]. Studies have shown that the administration of mAbs, including VHHs and their modifications, via the local routes is effective and even improves the potency of mAbs in contrast to systemic delivery [47,48,49,50,51]. Therefore, direct delivery of mAbs to the airway may be an appropriate approach for treatment and prevention of infection caused by influenza viruses.

In this study, we characterized the fusion particles of the aforementioned VHHs [35] with human IgG Fc domain—G2.3-Fc and H1.2-Fc. Both VHH-Fcs efficiently neutralized Group 1 influenza A viruses in vitro and in vivo. G2.3-Fc, compared to H1.2-Fc, exhibited a broader in vitro neutralizing activity, and protected mice against lethal H1N1 and H5N2 challenge in vivo with greater potency. Our findings support G2.3-Fc as a potential agent for both prophylaxis and treatment of Group 1 IAV infection.

## 2. Materials and Methods

### 2.1. Viruses and Recombinant Proteins

A/California/07/2009 (CA/09(H1N1)), A/Victoria/2570/2019 (VA/19(H1N1)), А/Duck/mallard/Moscow/4970/2018 (duck/MW/18(H1N1)), A/Mallard duck/Pennsylvania/10218/84 (duck/PA/84(H5N2)), A/Swine/Hong Kong/9/98 (swine/HK/98(H9N2)), and A/Black Duck/New Jersey/1580/78 (duck/NJ/78(H2N3)) were grown in embryonated hens’ eggs according to standard viral culture techniques. The mouse adapted (ma) A/California/07/2009 (CA/09(H1N1)ma) and A/Mallard duck/Pennsylvania/10218/84 (duck/PA/84(H5N2)ma) were obtained by serial lung-to-lung passages, as described elsewhere [52]. For ADCC and ADCP assays, recombinant serotype 5 adenovirus, Ad5-swH1opt/California, carrying a codon-optimized gene encoding HA A/California/07/2009 (H1N1) protein, was kindly provided by Dr. Maxim M. Shmarov (N.F. Gamaleya NRCEM, Moscow, Russia). Recombinant full-length HA (rFL HA) subtype H1 (A/California/04/2009) was purchased from Sino Biological (Beijing, China).

### 2.2. Cell Lines

The CHO-S cell line was obtained from Thermo Fisher Scientific (Waltham, MA, USA) cat. no. R80007. Caco-2, and A549 cell lines were purchased from the Russian collection of vertebrate cell lines (Saint Petersburg, Russia). Jurkat-Lucia™ NFAT-CD16 cells and Jurkat-Lucia™ NFAT-CD32 were obtained from InvivoGen (San Diego, CA, USA) cat. code jktl-nfat-cd16 and jktl-nfat-cd32, respectively.

### 2.3. VHH-Fc Fusion Construction, Expression and Purification

Nucleotide sequences coding the previously described VHHs, H1.2, and G2.3 [35] fused to the human IgG1 Fc-fragment and the Llama IgG2b hinge were synthetized (Evrogen, Moscow, Russia) and cloned into the pCEP4 mammalian expression vector (Thermo Fisher Scientific, USA). The sequences of the Fc-fragment (CH2 and CH3 domains) and hinge region are available in the GenBank database (accession numbers JQ666008.1 and AAX73259.1, accordingly). CHO-S cells were transfected with VHH-Fc containing construct using the CHO Gro System (Mirus Bio, WI, USA), according to the manufacturer’s protocol. Expressed antibodies were purified from a culture supernatant using HisTrap™ MabSelect™ Sure columns (Cytiva, Danaher Corporation, Washington, DC, USA). Purity was analyzed by sodium dodecyl sulfate polyacrylamide gel electrophoresis (SDS–PAGE).

As positive controls for enzyme-linked immunosorbent assay (ELISA), microneutralization assay (MN), ADCC and ADCP reporter assays, we used broadly neutralizing VHHs, SD38, and SD36, fused to hIgG1-Fc [28]. These VHH-Fcs were synthesized, expressed, and purified as described above. 

### 2.4. Sodium Dodecyl Sulphate-Polyacrylamide Gel Electrophoresis (SDS–PAGE)

SDS–PAGE was performed as described elsewhere. Proteins were separated using Any kD™ Mini-PROTEAN^®^ TGX™ Precast Protein Gels (Bio-Rad, Hercules, CA, USA) in Tris-Glycine buffer. Samples were prepared as follows: for non-reducing, SDS–PAGE samples were mixed with an equal volume of 2× Laemmli sample buffer and loaded into gel lanes; for reducing, SDS–PAGE samples were heated at 95 °C for 10 min with 2× Laemmli buffer containing 2-mercaptoethanol. As protein molecular weight standard, Precision Plus Protein™ Unstained Protein Standards (Bio-Rad, Hercules, CA, USA) was used. Gel Doc™ EZ imaging system (Bio-Rad, Hercules, CA, USA) was used for stain-free gels visualization.

### 2.5. Enzyme-Linked Immunosorbent Assay (ELISA)

Polystyrene microplates (Corning Inc., Corning, NY, USA) were coated overnight at 4 °C with 1 µg/mL rFL HA in carbonate-bicarbonate buffer pH 9.6, then washed three times with PBS containing 0.05% Tween-20 (TPBS), and incubated for 1 h with blocking buffer (TPBS containing 1% *w*/*v* casein) at 37 °C. After that, plates were rinsed three times with TPBS and VHH-Fc (serial dilutions in blocking buffer) were added to the wells and incubated for 1 h at 37 °C. Immunoplates were washed four times and bound antibodies were detected using polyclonal goat anti-human IgG HRP-conjugated antibodies (MilliporeSigma, Burlington, MA, USA), diluted 1:20,000 in blocking buffer. After five washes, 3,3′5,5′-tetramethylbenzidine (TMB) (Bio-Rad, Hercules, CA, USA) was added as a substrate. Fifteen minutes later, the reaction was stopped by the addition of 1 M H_2_SO_4_ and the absorbance was read at 450 nm. The half maximal effective concentration (EC_50_) values were calculated using four-parameter logistic regression using GraphPad Prism 7 (GraphPad Software Inc., San Diego, CA, USA).

The antibody binding activity to untreated and denatured HA was measured as described above. rFL HA subtype H1 was denatured in 0.1% SDS, 50 mM DTT by heating at a dry block thermostat at 99 °C for 10 min.

### 2.6. In Vitro HA Cleavage Assay

The procedure was performed as described previously [53], with minor modifications. rFL HA H1 (0.5 µg) was incubated with VHH-Fc (0.5 µg) for 1 h at room temperature, and then 50 ng of N-tosyl-L-phenylalanine chloromethyl ketone-treated trypsin (TPCK-trypsin) was added. The mixture was incubated for 10 min at 37 °C. The samples were heated with Laemmli sample buffer with 2-mercaptoethanol at 95 °C for 20 min and separated on SDS–PAGE using Any kD™ Mini-PROTEAN^®^ TGX™ Precast Protein Gels (Bio-Rad, Hercules, CA, USA).

### 2.7. Low-pH-Induced Conformational Changes ELISA

The procedure was carried out as described previously, with minor modifications [53]. 1 µg/mL rFL HA in carbonate–bicarbonate buffer pH 9.6 was immobilized on the surface of 96-well polystyrene microplates (Corning Inc., Corning, NY, USA) overnight at 4 °C. The plates were washed three times with TPBS and incubated for 1 h with blocking buffer at 37 °C and 350 rpm. Afterward, plates were rinsed three times with TPBS. TPCK-treated trypsin (25 ng/mL) was added after incubation for 1 h at 37 °C, and the plates were washed again. Citrate buffer 0.1 M with the addition of 150 mM NaCl with varying pH (7.4, 6 and 5) was added to the wells, and immunoplates were incubated for 1 h at room temperature without shaking. Plates were washed three times and VHH-Fcs diluted in blocking buffer were added. The conventional ELISA protocol was followed as described above.

### 2.8. Microneutralization (MN) Assay

H1.2 and G2.3 in monovalent VHH format for MN assays were expressed and purified as described previously [35]. Caco-2 cells were maintained in complete Dulbecco’s Modified Eagle Medium (DMEM) supplemented with 20% fetal bovine serum (FBS). Caco-2 cells were seeded in 96-well culture plates (4 × 10^4^ cells/well) the day before the experiment. Two-fold serially diluted VHHs or VHH-Fcs were mixed with an equal volume of virus (100 TCID_50_/well) in Medium 199 with Hanks′ salts (MilliporeSigma, USA) and incubated at 37 °C for 40 min. Caco-2 cells were washed twice with DMEM without FBS, and samples were added and incubated with the cells at 37 °C for 40 min. After incubation, the medium was replaced with a fresh medium and plates were incubated at 37 °C with 5% CO_2_ for 96 h. The assay was performed in quadruplicate. Each 96-well plate contained positive (virus-inoculated cells) and negative (mock-inoculated cells) controls, as well as the control SD38-Fc. Neutralization of the virus was confirmed by hemagglutination assay. The cell medium was mixed with an equal volume of 0.75% chicken erythrocytes and incubated for 30 min at room temperature. Negative (PBS with erythrocytes) and positive (8 hemagglutinating units of virus with erythrocytes) controls were included in every plate. A button formation was scored as absence of hemagglutination, indicating complete neutralization of the virus by the tested antibody. The minimal neutralizing concentration was defined as the lowest antibody concentration that resulted in the absence of agglutination. Half-maximal inhibitory concentration (IC_50_) values were calculated using the Reed and Muench method [54].

### 2.9. In Vitro ADCC and ADCP Reporter Assay

A549 cells were maintained in DMEM supplemented with 10% inactivated FBS. A549 cells were seeded in 96-well culture plates 2 × 10^4^ cells/well on the day of the experiment. After the cell adhesion, culture media was replaced with the medium containing Ad5-swH1opt/California (15 PFU/cell) and plates were incubated in a 5% CO_2_ incubator at 37 °C for 72 h. After incubation, the cells were washed once with a fresh medium, and VHH-Fcs diluted in the same medium were added to the wells. The plates were incubated in a 5% CO_2_ incubator at 37 °C for 1 h, then the cells were washed once to remove unbinding VHH-Fc. The reporter cells Jurkat-Lucia™ NFAT-CD16 cells or Jurkat-Lucia™ NFAT-CD32 were added and the experiment was performed as described previously [55]. The analysis included positive control (SD38-Fc), negative control (corresponding VHHs without Fc-fragment and non-relevant VHH-Fc), and intact cells. The assay was performed in quadruplicate.

### 2.10. Prophylactic and Therapeutic Efficacy Studies in Mice

All animal procedures were approved by the Institutional Ethics Committee on Experimental Animals (protocol #19 dated 2 March 2022). Specific-pathogen-free female 6–8-week-old BALB/c mice were inoculated intranasally with 5 LD_50_ of mouse-adapted A/California/07/2009 (H1N1) or A/Mallard duck/Pennsylvania/10218/84 (H5N2) (50 µL, divided equally between the nostrils), observed daily for clinical signs, and weighed. Antibodies were diluted in phosphate-buffered saline (PBS) and administered intranasally or intraperitoneally in a volume of 50 µL or 200 µL, respectively. Mice were anesthetized by inhaled anesthetic isoflurane before intranasal administration of VHH-Fc or challenge virus. Depending on the experiment, antibodies were administered up to 24 h before or after challenge, as specified in the figure legends. A 25% loss in body weight was determined as the clinical endpoint at which the agonizing mice were humanely euthanized.

In order to determine the effect of antibody delivery on lung virus titer production, mice were administered i.p. with 3 mg/kg VHH-Fc, then, 24 h later, challenged with 5 LD_50_ of virus, and euthanized 3 days after challenge. Lung homogenates were prepared in 1 mL Medium 199 with Hanks′ salts, cleared by centrifugation at 4 °C, and used for virus titration, as described above. Lung viral titers were expressed in TCID_50_ per milliliter and determined using the Reed and Muench method [54].

### 2.11. Multiple Sequence Alignment and Phylogenetic Tree

Sequences of the FL HA protein of CA/09(H1N1), VA/19(H1N1), duck/MW/18(H1N1), duck/PA/84(H5N2), duck/NJ/78(H2N3), and swine/HK/98(H9N2) were downloaded from the Influenza Research Database (IRD) or the Nucleotide database (NCBI) and aligned using the MEGA X Software (Penn State University, State College, PA, USA) with the MUSCLE method. The phylogenetic tree was produced using the maximum likelihood method and visualized in the MEGA X program.

### 2.12. Statistical Analysis

All statistical analyses and plot generation were conducted using Prism 7 Software (GraphPad Software, Inc., USA). EC_50_ values were determined using four-parameter logistic regression. TCID_50_ and IC_50_ values were calculated using the Reed and Munch method. Differences between mice groups were analyzed using the nonparametric Kruskal–Wallis test (one-way ANOVA), followed by Dunnett’s multiple comparisons test. The Mantel–Cox log-rank test was used to assess the statistical significance of Kaplan–Meier survival curve comparisons.

## 3. Results

### 3.1. Construction and Characterization of VHH-Fc

**Figure 1:**
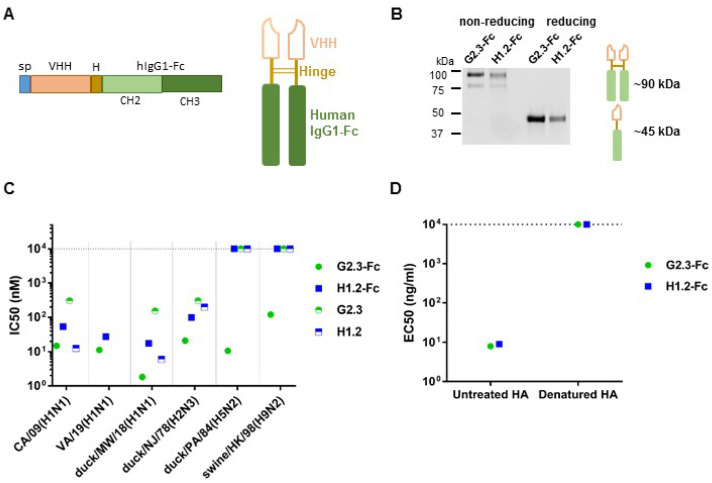
Characterization of G2.3-Fc and H1.2-Fc. (**A**) A schematic representation of the VHH-Fc constructs. sp—signal peptide, H—hinge, CH2 and CH3—heavy chain constant domains of hIgG1-Fc. (**B**) Analysis of purified VHH-Fc using SDS–PAGE. Theoretical molecular weights for VHH-Fc under reducing and non-reducing conditions are ~45 kDa and 85–90 kDa, respectively. (**C**) Neutralizing activity of VHH and VHH-Fc in vitro against Group 1 IAV. The neutralizing potency of VHH against influenza VA/19 (H1N1) virus was not tested. IC_50_ is expressed in nanomolar concentration and was measured in quadruplicate. (**D**) EC_50_ values determined in ELISA show binding of VHH-Fc to untreated and denatured rFL HA (A/California/04/2009). EC_50_ values are expressed in nanogram per milliliter. Dotted lines indicate the absence of activity.

In a previous study, we described anti-HA stem single-domain antibodies, G2.3 and H1.2, which showed potent neutralizing activity in vivo against H1N1 and H5N2 IAV [35]. In this study, we modified these VHH with human IgG1 Fc-fragment in order to obtain prolonged serum half-life and to engage Fc-effector functions (Figure 1A). Purified antibodies were analyzed in SDS–PAGE, and the formation of dimeric Fc-fusion VHHs of approximately 85–90 kDa was confirmed (Figure 1B). The HA-binding activity of VHH-Fc was determined in ELISA using rFL HA (A/California/04/2009) as antigen (Supplementary Figure S1).

In order to determine the neutralizing potency of our antibodies, we performed a MN assay with the H5N2, H2N3, and H9N2 influenza viruses, and different strains of H1N1 subtype (Figure 1C and Figure S2). We compared the activity of Fc-fused and monovalent forms of VHH to explore whether a shift to a bivalency of VHH affected their neutralizing performance. We observed that G2.3-Fc, compared to its monovalent form, not only improved potency for H1 and H2 subtypes, but also obtained the ability to neutralize H5 and H9 subtypes. We detected an 80-fold increase in neutralizing activity in the case of duck/MW/18(H1N1) virus: IC_50_ values for monomeric G2.3 and Fc-modified molecule were 152 nM and 1.82 nM, respectively (Supplementary Figure S2B). It was shown that in contrast to the G2.3-Fc antibody, the H1.2-Fc exhibited lower neutralizing potency against H1 and H2 viruses and did not neutralize H5 and H9 viruses in vitro. It should be noted that reformatting H1.2 VHH into a bivalent format decreased its in vitro potency. We speculate that it might be a result of steric hindrance [56,57].

In order to define whether the binding of G2.3-Fc and H1.2-Fc to H1 HA protein was conformation-dependent, we performed ELISA with denatured HA (Figure 1D). The antibodies lost their ability to bind to HA after denaturation, but maintained binding activity with native HA protein. ELISA studies showed that both VHH-Fcs recognize conformational epitopes on the surface of HA protein.

**Figure 2:**
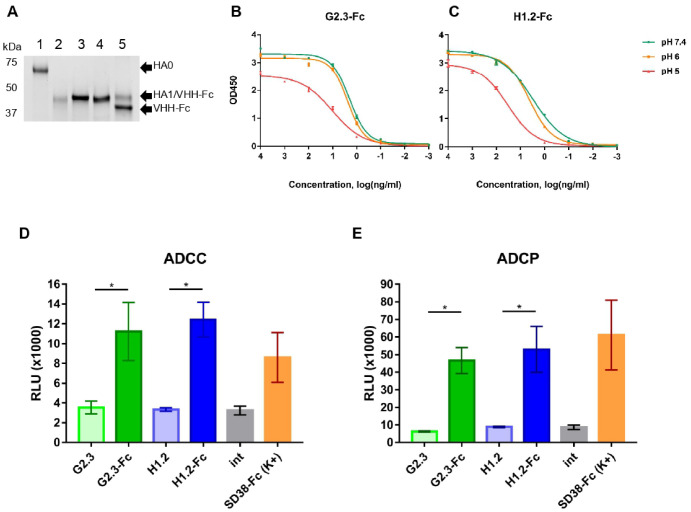
Mechanism of antiviral action of VHH-Fc. (**A**) Cleavage inhibition assay: rFL HA H1 was pre-incubated with VHH-Fc and then cleaved with TPCK-trypsin. 1—rFL HA H1 (~65–67 kDa) under reducing conditions; 2—product of rFL HA H1 tryptic digestion: HA1 (~45 kDa), HA2 band is not visible; 3—rFL HA H1 preincubated with G2.3-Fc, HA1 band overlaps with the single chain of VHH-Fc band due to similar MW; 4—rFL HA H1 preincubated with H1.2-Fc, HA1 band overlaps with the single chain of VHH-Fc band; 5—rFL HA H1 preincubated with control anti-HA single-domain antibody, SD38-Fc, HA1 band separates from the single chain of VHH-Fc band. (**B**,**C**) ELISA with rFL HA H1 subjected to trypsinization and treated with different pH (citrate buffer pH 7.4, 6 and 5). (**D**,**E**) Evaluation of Fc-mediated functions, ADCC, and ADCP: the effector cells, featuring CD16 (ADCC, (**D**)) or CD32 (ADCP, (**E**)) were added to A549 cells, expressing HA A/California/07/2009 (H1N1) protein and previously incubated with 250 nM of VHH or VHH-Fc. ADCC and ADCP activities were expressed as RLU—relative luminescence units. Error bars represent means ± SD. Asterisks indicate significant differences: *, *p* = 0.0286. int—intact cells.

In order to understand the potential mechanism underlying the neutralizing activity, we explored the influence of the antibodies on the membrane fusion process. This process consists of two stages, which can be affected by antibodies: HA cleavage by host-cell trypsin-like proteases and low-pH–induced conformational change of HA in endosomes. As control antibodies, we used two previously described anti-HA single-domain antibodies, VHH-Fc, SD38-Fc, and SD36-Fc, with different binding profiles [28]. Initially, we modeled the proteolytic activation of HA with TPCK-trypsin in the presence of VHH-Fc. The results showed that both VHH-Fcs, the same as control antibody SD38-Fc, could not prevent protease cleavage of HA (Figure 2A). Afterwards, to test whether G2.3-Fc and H1.2-Fc could bind to HA, which undergoes low-pH-induced conformational change, we subjected rFL HA H1 to proteolytic activation, then treated cleaved HA with low pH and followed the conventional ELISA protocol. ELISA studies showed that both G2.3-Fc and H1.2-Fc maintain their binding activity at pH 7.4 and pH 6, while with decreasing pH to 5, the activity of VHH-Fc slightly declines (Figure 2B,C). In comparison with the control antibody SD38-Fc, affinity of G2.3-Fc and H1.2-Fc in acidic pH was lower (Supplementary Figure S3A). In contrast, the attachment of another previously described antibody SD36-Fc decreased markedly with a decrease in the pH (Supplementary Figure S3B), which is consistent with the results obtained by the Laursen et al. [28]. We speculate that the potential mechanism of antiviral action of G2.3-Fc and H1.2-Fc may lie in inhibiting low-pH–induced conformational changes that would block membrane fusion.

At the next stage, we evaluated the effector functions of VHH-Fc, since previous studies indicate a protective role for Fc-mediated functions of stem-specific antibodies against influenza infection in vivo [40,41,42,58]. In order to assess the possibility of FcγR activation by our VHH-Fc, we performed ADCC and ADCP reporter assays. In both ADCC and ADCP tests, the activity of G2.3-Fc and H1.2-Fc was statistically significant compared to monovalent VHH and intact cells, and they showed similar to SD38-Fc potency (Figure 2D,E). These data showed that fusion to Fc domain resulted in the acquisition of effector mechanisms, and that VHH-Fc could effectively engage Fc-dependent functions.

### 3.2. Prophylactic Efficacy of VHH-Fc Administered Systemically or Locally against H1N1 and H5N2 IAV

**Figure 3:**
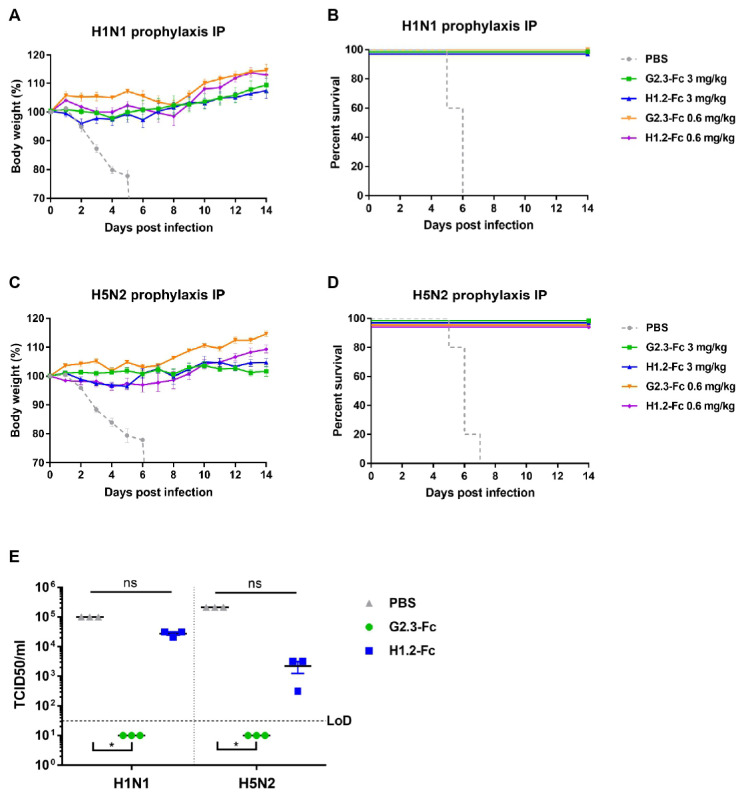
Prophylactic efficacy of systemically administered VHH-Fc in mice. Body weight changes and survival curves of BALB/c mice (n = 5 per group), treated via the i.p. route with G2.3-Fc or H1.2-Fc at different doses 24 h before infection with influenza CA/09(H1N1)ma virus (**A**,**B**) or duck/PA/84(H5N2)ma virus (**C**,**D**). As a negative control, a vehicle (PBS) was administered 24 h before infection via the i.p. route. Body weight curves represent mean value ± SEM. (**E**) Lung viral titers following 3 mg/kg G2.3-Fc or H1.2-Fc prophylaxis and H1N1 or H5N2 virus challenge in BALB/c mice. Lung viral titers are expressed in TCID_50_ per milliliter. Error bars represent means ± SEM. Asterisks indicate significant differences from the control group (*, *p* = 0.0105; ns—non-significant).

We initially evaluated the effect of systemic delivery for both Fc-fused antibodies (Figure 3). Mice were injected i.p. with 3 or 0.6 mg/kg of VHH-Fc 24 h before infection with 5 LD_50_ of CA/09(H1N1)ma or duck/PA/84(H5N2)ma virus. All mice administered 3 mg/kg of G2.3-Fc survived both the H1 and H5 virus challenges (Figure 3B,D) and did not demonstrate any weight loss (Figure 3A,C). Mice that received H1.2-Fc (3 mg/kg) also survived the H1 and H5 virus challenges (Figure 3B,D), but exhibited transient weight loss with recovery by day 7 post-infection (Figure 3A,C). In contrast, all control mice treated with the vehicle (PBS) lost weight and either succumbed to infection or reached a humane endpoint (25% weight loss) and were euthanized by day 7 post-infection. Surprisingly, the administration of as low as 0.6 mg/kg of both VHH-Fc showed full protection against both viruses; antibody-treated mice exhibited no or transient weight loss (in case of H1.2-Fc prophylaxis) with recovery by day 9 post-infection. In the control groups, mice challenged with H1N1 and H5N2, respectively, succumbed to infection or reached the endpoint by day 7 post-infection.

In order to evaluate the effect of systemic antibody delivery on lung virus titer production, mice were treated with 3 mg/kg of VHH-Fc 24 h before infection with 5 LD_50_ of CA/09(H1N1)ma or duck/PA/84(H5N2)ma virus. On day 3 post-infection, mice were euthanized and lungs were harvested to determine viral titers. Prophylactic i.p. administration of G2.3-Fc reduced lung viral titers compared with the groups that received the vehicle (Figure 3E). The lung viral titers in all groups treated with G2.3-Fc were undetectable, whereas the administration of H1.2-Fc did not lead to a statistically significant reduction in H1N1 and H5N2 virus titers. Overall, the results are consistent with the absence of clinical symptoms in G2.3-Fc–treated mice.

**Figure 4:**
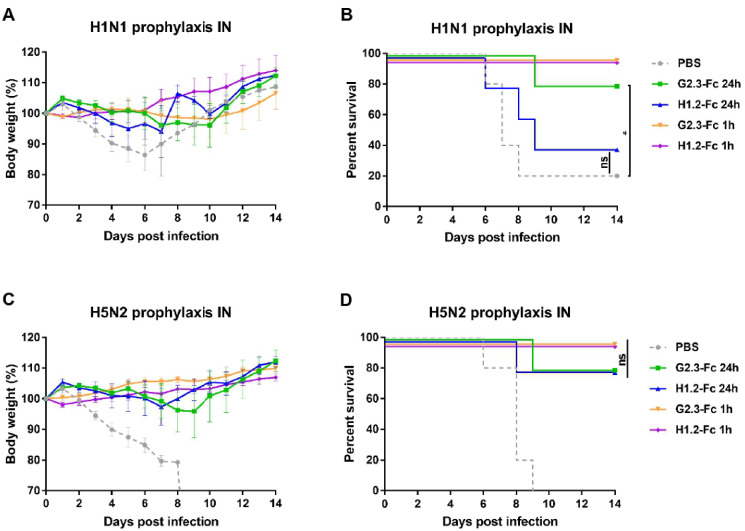
Prophylactic efficacy of locally administered VHH-Fc in mice. Body weight changes and survival curves of mice (n = 5 per group), treated via the i.n. route with G2.3-Fc or H1.2-Fc in a dose of 0.6 mg/kg 24 h or 1 h before infection with influenza CA/09(H1N1)ma virus (**A**,**B**) or duck/PA/84(H5N2)ma virus (**C**,**D**). As a negative control, a vehicle (PBS) was administered 1 h before infection via the i.n. route. Body weight curves represent mean value ± SEM. Significant differences between groups are shown: *, *p* = 0.033; ns—non-significant.

Based on early studies that showed improved in vivo efficacy of airway antibody delivery compared with systemic delivery [48,49], we performed a prophylactic administration of VHH-Fc via the intranasal route. Mice were initially treated 0.6 mg/kg of G2.3-Fc or H1.2-Fc, and then were challenged 24 h or 1 h later with 5 LD_50_ of CA/09(H1N1)ma or duck/PA/84(H5N2)ma virus. The survival and weight loss results for mice challenged with H1N1 are shown in Figure 4A,B. Mice treated with the vehicle control lost weight rapidly and 4 of 5 mice succumbed to infection or reached the endpoint by day 8. All mice treated with antibodies 1 h before H1N1 infection survived the lethal challenge without transient weight loss. Administration of VHH-Fc 24 h before infection resulted in reduced protection compared with those treated 1 h before the challenge. Mice that were inoculated i.n. with G2.3-Fc 24 h before H1N1 challenge showed 80% survival rate and a weight loss of 5% of their initial body weight, while only 40% of mice that received H1.2-Fc survived. In the context of the H5N2 challenge (Figure 4C,D), all PBS control animals lost weight and succumbed to infection by day 9. Mice treated with VHH-Fc 1 h before challenge were fully protected from death, weight loss, and clinical symptoms. Nevertheless, when the infection occurred 24 h after antibody treatment, 80% mice survived; the difference between groups who received VHH-Fc 1 h and 24 h before infection was statistically insignificant (*p* > 0.05).

Thus, obtained results indicated that G2.3-Fc and H1.2-Fc could protect mice against H1N1 and H5N2 viruses in vivo. Prophylaxis with 0.6 mg/kg of G2.3-Fc is effective against both H1 and H5 viruses when administered via either a systemic or local route.

### 3.3. Therapeutic Efficacy of VHH-Fc Administered Systemically or Locally against H1N1 and H5N2 IAV

**Figure 5:**
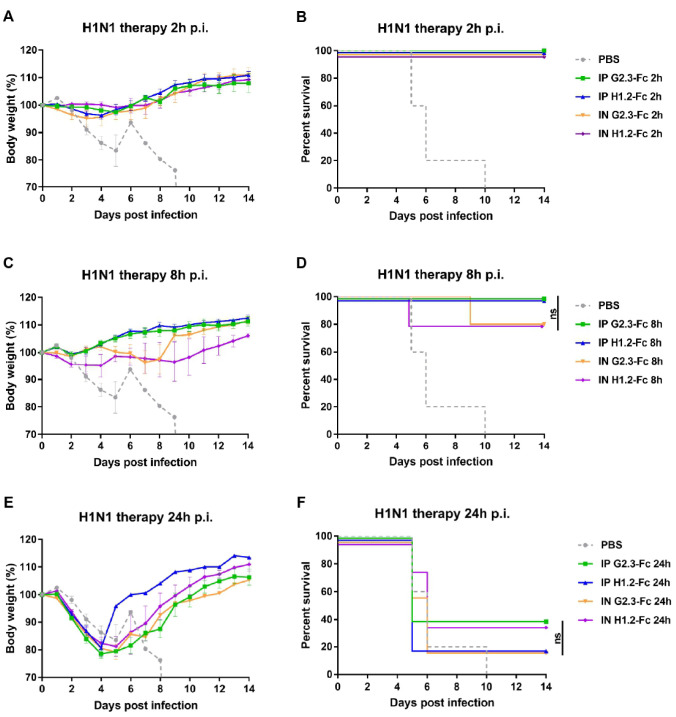
Therapeutic efficacy of VHH-Fc against H1N1 IAV in BALB/c mice. Body weights and survival curves of mice (n = 5 per group) treated with G2.3-Fc or H1.2-Fc with a dose of 10 mg/kg in case of i.p. injection or 2 mg/kg when delivered via the i.n. route. VHH-Fc were administered 2 h (**A**,**B**), 8 h (**C**,**D**), or 24 h (**E**,**F**) after the infection with 5 LD_50_ of influenza CA/09(H1N1)ma virus. As a negative control, a vehicle (PBS) was administered 2 h after the infection via the i.n. route. Body weight curves represent mean value ± SEM. ns—the difference between groups is non-significant.

**Figure 6:**
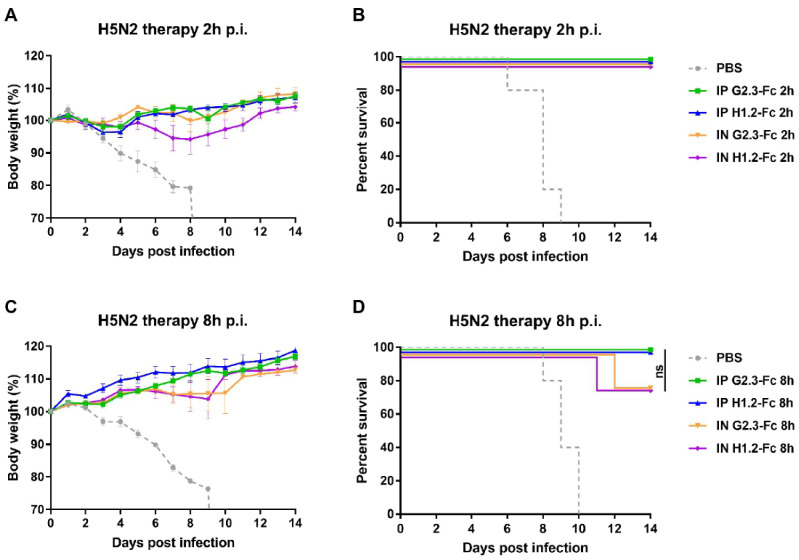
Therapeutic efficacy of VHH-Fc against H5N2 IAV in BALB/c mice. Body weights and survival curves of mice (n = 5 per group), treated with G2.3-Fc or H1.2-Fc with a dose of 10 mg/kg in case of i.p. injection or 2 mg/kg when delivered via the i.n. route. VHH-Fc were administered 2 h (**A**,**B**) or 8 h (**C**,**D**) after the infection with 5 LD_50_ of influenza duck/PA/84(H5N2)ma virus. As a negative control, a vehicle (PBS) was administered 2 h (**A**,**B**) or 8 h (**C**,**D**) after the infection via the i.n. route. Body weight curves represent mean value ± SEM. ns—the difference between groups is non-significant.

After observing the efficacy of systemic and local delivery of VHH-Fc in a prophylactic regimen at low doses, we investigated the therapeutic effectiveness of G2.3-Fc and H1.2-Fc when administered by different routes (Figure 5 and Figure 6). Mice were challenged with 5 LD_50_ of CA/09(H1N1)ma virus or duck/PA/84(H5N2)ma virus, and 2 h, 8 h, or 24 h later received VHH-Fc via i.p. or via i.n. route. A control group was inoculated i.n. with the vehicle (PBS) 2 h post-infection.

While testing the therapeutic efficiency, G2.3-Fc could completely protect mice with similar efficacy at a dose of 2 mg/kg, delivered locally, and 10 mg/kg, delivered systemically, when administered 2 h (Figure 5A,B and Figure 6A,B) or 8 h (Figure 5C,D and Figure 6C,D) after the challenge with both H1 and H5 influenza viruses. Mice treated with G2.3-Fc had no signs of respiratory distress and exhibited no or transient weight reduction of only 3% of their initial body mass. In the context of H1.2-Fc, the results were similar to that obtained for G2.3-Fc, but a slightly greater weight loss was observed, up to 6%, when H1.2-Fc was administered 2 h post-infection with H5N2 virus (Figure 6A,B). Unfortunately, a 24-h delay in a single administration of VHH-Fc after the challenge with H1N1 influenza virus did not show significant protection, although 2 of 5 mice survived in the groups treated i.p. with 10 mg/kg G2.3-Fc and i.n. with 2 mg/kg H1.2-Fc (Figure 6C,D). All mice in the control group and those treated 24 h post-infection experienced rapid and strong weight loss, but the surviving mice regained or even exceeded their initial body weight.

Overall, these findings demonstrate that G2.3-Fc and H1.2-Fc could therapeutically protect mice from H1N1 and H5N2 IAV; furthermore, i.n. administration of 2 mg/kg of VHH-Fc had the similar potency compared to the same VHH-Fc delivered via the i.p. route at a dose of 10 mg/kg.

## 4. Discussion

Influenza A viruses (IAV) lead to the death of hundreds of thousands of people in annual epidemics worldwide, and can initiate a pandemic; therefore, they remain a major public health problem. Among the Group 1 IAV, only H1 and H2 subtypes have caused pandemics, and due to their rapid mutations in HA, this is still a possibility [59,60]. Additionally, recent outbreaks of the H5 and H9 subtypes with high mortality rates have made them a significant pandemic threat [61,62]. Thus, it is an urgent necessity to design therapeutics against influenza infection, and broadly neutralizing antibodies can be a useful tool for this purpose. Herein, we describe a subtype cross-reactive VHH-Fcs, which were generated based on our previously reported VHHs. G2.3 and H1.2 showed potent neutralizing activity in vivo against H1N1 and H5N2 IAV [35].

In the context of influenza viruses, the antibody’s Fc-effector functions play a critical role in protection against infection and disease [41,42,63]. It has been reported that HA stem-specific cross-reactive antibodies provide Fc-dependent immunity, while strain-specific HA head antibody protection is Fc-independent [40,41,42,44,58]. The inclusion of the Fc-domain to the VHH structure not only adds Fc-effector functions, but also increases serum half-life [64,65] and improves the binding ability of single-domain antibodies by bivalency-imparting [29,66,67]. Fc-fragment of human IgG1 is the isotype of choice for mAb-based therapeutic, as it effectively binds to C1q and different Fc-receptors, thus providing a cell-mediated mechanism required for viral elimination. A recent study of broadly-neutralizing VHHs against influenza confirmed a significant increase in in vivo potency and neutralization breadth for Fc-fused VHHs [28]. In our work, two anti-HA stem VHHs, H1.2 and G2.3, were linked to human IgG1-Fc to add them effector functions and retard their clearance.

The neutralization breadth of VHH-Fc was studied in vitro in a microneutralization assay on Caco-2 cells. G2.3-Fc exhibited greater neutralizing activity than H1.2-Fc, being able to neutralize all tested subtypes of Group 1 IAV, including H1 strains isolated from different species, H2, H5, and H9, with IC_50_ values similar to those of previously described cross-neutralizing VHH-Fcs and mAbs [28,68,69,70]. Fusion to the Fc-fragment not only increased the neutralizing potency of G2.3-Fc against H1 and H2 subtypes, but also allowed the antibody to effectively inhibit the growth of H5 and H9 subtypes compared to monovalent G2.3, which is consistent with the previously published studies [28,29,30]. Surprisingly, conversion of H1.2 into bivalent Fc-fusion form did not achieve the same results. We speculate that this might be a result of steric hindrance [56,57] and that spatial interference probably results in a reduced neutralizing activity of VHH-Fc, compared to the small-sized VHH domain. The dense arrangement of HA on the surface of the virion may hinder access to the stem domain for larger Fc-fused molecules to varying degrees, depending on the specific antibody epitope.

Many anti-stem broadly-neutralizing antibodies block viral entry by inhibition of the membrane fusion, thus providing the neutralizing effect in vitro [68,69,71]. Membrane fusion consists of two processes: proteolytic activation of HA and pH-induced conformational change in endosomes. Neither G2.3-Fc nor H1.2-Fc could prevent the protease cleavage of HA. Though, given the fact that both G2.3-Fc and H1.2-Fc could bind to low-pH-treated conformation of H1 HA, we speculate that the possible antiviral mechanism of VHH-Fc lies in inhibiting low-pH-induced conformational changes. Identifying epitopes in the HA protein for our VHH-Fc could further shed light on the mechanism of action.

The results of this study revealed that administration of both VHH-Fc can effectively protect mice against lethal H1N1 and H5N2 challenges when administered a prophylaxis regimen either via a systemic or local route. A dose as low as 0.6 mg/kg of both G2.3-Fc and H1.2-Fc was sufficient to protect mice from disease and death with H1N1 or H5N2 IAV. To the best of our knowledge, this is the lowest effective prophylactic dose of any anti-influenza HA antibody. Moreover, administration of G2.3-Fc reduced the viral loads in the lungs below limits of detection. Considering that the influenza viruses mainly affect the respiratory tract, it could be beneficial to deliver therapeutic antibodies directly to the site of infection [46]. The bioavailability of antibodies administered locally exceeds systemic administration, which allows a reduction in the amount of treatment and, accordingly, in its cost [48,49]. The undeniable advantage of the local administration of antibodies is not only a reduction in the overall dose, but additionally a more convenient application and diminishing the risks associated with intravenous drug delivery [72]. Therefore, our next step was to determine whether the prophylactic intranasal inoculation of VHH-Fc had comparable protection against H1 and H5 IAV challenges as i.p. administration. We observed that 0.6 mg/kg of G2.3-Fc delivered 24 h or 1 h before infection was required for statistically significant protection of the mice challenged with either H1N1 or H5N2 IAV. The fact that i.n. administration of VHH-Fc 24 h before infection protected 80% of mice may be a result of the following: (i) short half-life in the respiratory tract, which is only about 22 h for IgG [73]; and (ii) little or no influence of Fc-dependent functions [49,73]. The subsequent pharmacokinetic studies of these VHH-Fcs are needed.

Our study showed that both G2.3-Fc and H1.2-Fc can protect mice in the therapeutic mode when delivered via i.n. or i.p. route, up to 8 h after the infection. Given the results of previous studies on the increased effectiveness of antibodies when delivered locally [48,49,74], we initially took a lower dose for i.n. administration of VHH-Fc. The dose of 2 mg/kg delivered i.n. resulted in a similar protection as 10 mg/kg dose administered via a systemic route. We did not investigate the effect of the local administration of VHH-Fc in lower doses, however, according to previous studies, it is possible to improve the potency of locally administered antibody up to 10-fold greater that the same antibody exhibits when delivered via a systemic route [48,49]. Although therapy with a single dose of VHH-Fc 24 h post-infection did not offer complete protection against H1N1 infection, surviving mice gained weight and even exceeded their initial body mass up to the end of the experiment. Reduced potency of antibodies in the postexposure regimen is time-dependent, and may be associated with the larger amount of infectious viruses produced in the lungs after replication cycles [75] or with rapidly decreased VHH-Fc concentration in the result of formation and removal of immune complexes [76,77]. An improvement in the therapeutic effectiveness of VHH-Fc can be achieved by increasing the amount of delivered antibody, due to multiple injections with a certain interval or by obtaining an antibody cocktail [74,78,79,80,81]. In future studies, we will explore whether these methods could enhance the potency of our VHH-Fc.

Interestingly, H1.2-Fc could not ensure the neutralization of H5 subtype in vitro, notwithstanding the in vivo efficacy against H5 IAV. It should be noted that the protective potency of antibodies in vivo also depends on the Fc-mediated mechanisms, including ADCC, ADCP, and CDC (complement dependent cytotoxicity) [39,42,58,82]. Thus, antibodies lacking neutralizing activity in vitro can prevent infection in vivo through a cell-mediated mechanism by inhibiting virus release from infected cells as well as cell-to-cell spreading of viral progeny [83,84,85,86,87,88]. We have shown that both G2.3-Fc and H1.2-Fc have ADCC and ADCP activity in vitro, thus suggesting that G2.3-Fc and H1.2-Fc could engage Fc-effector functions for in vivo protection. Whether VHH-Fc could prevent cell-to-cell viral spread needs further investigation.

We have previously shown that as far as 1 h after injection of the monomeric form of VHH, only about 10% or even less of the initial concentration remains in the blood of experimental animals, and the VHH-Fc modification greatly increased their neutralizing potency and serum half-life [38]. Our data were in agreement with the reports on other VHHs [89,90]. Even though in some cases, such as imaging applications, rapid clearance of substances is of interest, for systemic prophylactic or therapeutic applications, elongated serum half-life is preferable. For this reason, the in vivo potency of monomeric VHH was not evaluated in this study.

In conclusion, we developed two Fc-fused sdAbs (G2.3-Fc and H1.2-Fc), which showed cross-protection against Group 1 IAV in prophylactic and therapeutic regimens. This study reported that the efficacy of local administration of antibodies is on par with systemic delivery in the context of influenza infection. Local passive immunotherapy via the mucosal route allows self-administration to patients and reduces the risks associated with injectable drug delivery. These VHH-Fc can find clinical application as preventive treatment and emergency therapies for IAV infection. The findings of this study could provide an effective strategy for influenza prevention and treatment.

## Data Availability

The data are contained within the article and supplemental figures.

## Supplementary Materials

The following supporting information can be downloaded at: https://www.mdpi.com/article/10.3390/v14112485/s1, Figure S1: Determining of HA-binding activity of VHH-Fc in ELISA; Figure S2: Influenza A viruses used in this study, their phylogenetic analysis and results of microneutralization assay; Figure S3: Low pH-induced conformation changes ELISA; Figure S4: Original image of SDS-PAGE from Figure 1B and Figure 2A.
