# Supplementary material for: Cross-Reactive Fc-Fused Single-Domain Antibodies to Hemagglutinin Stem Region Protect Mice from Group 1 Influenza a Virus Infection
Source: Viruses. 2022 Nov 10;14(11):2485. doi: 10.3390/v14112485 (PMC9698552; doi:10.3390/v14112485)
Supplement: Supplementary file 1 [file viruses-14-02485-s001.zip › viruses-1982780-supplementary.pdf]

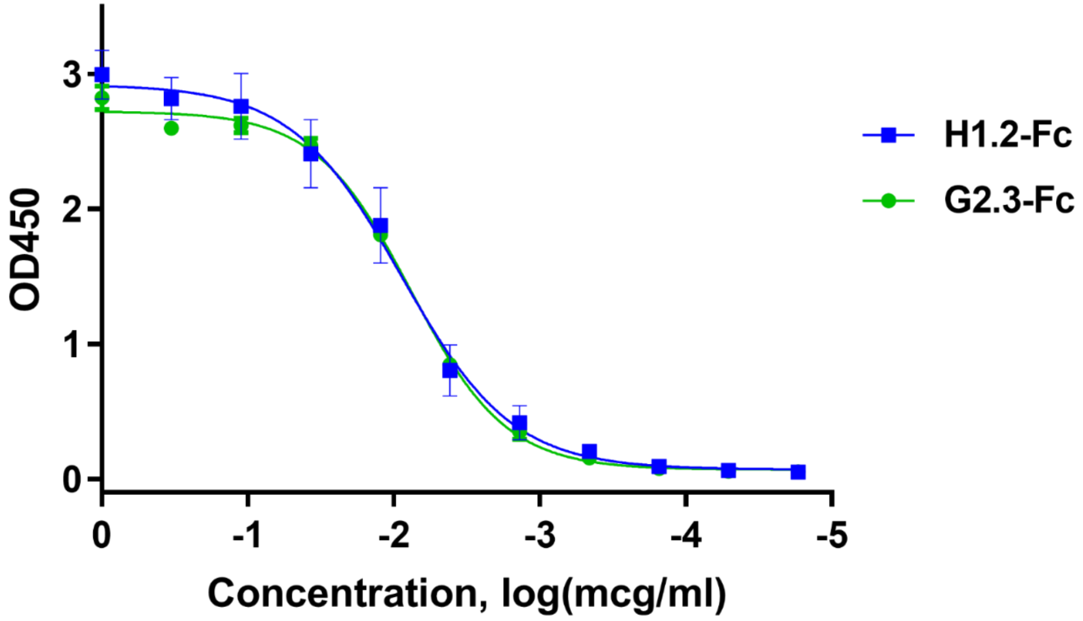

Figure S1: Determining of HA-binding activity of VHH-Fc in ELISA. 100 ng/well of rFL HA (A/California/04/2009) was used as antigen, EC_{50} was 8 ng/mL for G2.3-Fc and 9 ng/mL for H1.2-Fc.

A

Influenza virus	Abbreviation	Subtype	Host	Phylogenetic clade
A/California/07/2009 (H1N1)	CA/09(H1N1)	H1N1	human	H1
A/California/07/2009 (H1N1) ma	CA/09(H1N1)ma	H1N1	human	H1
A/Victoria/2570/2019 (H1N1)	VA/19(H1N1)	H1N1	human	H1
A/Duck/mallard/Moscow/4970/2018 (H1N1)	duck/MW/18(H1N1)	H1N1	avian	H1
A/Mallard duck/Pennsylvania/10218/84 (H5N2)	duck/PA/84(H5N2)	H5N2	avian	H1
A/Mallard duck/Pennsylvania/10218/84 (H5N2) ma	duck/PA/84(H5N2)ma	H5N2	avian	H1
A/Black Duck/New Jersey/1580/78 (H2N3)	duck/NJ/78(H2N3)	H2N3	avian	H1
A/Swine/Hong Kong/9/98 (H9N2)	swine/HK/98(H9N2)	H9N2	swine	H9

B

Virus	IC50 (nM)			
	G2.3	G2.3-Fc	H1.2	H1.2-Fc
CA/09(H1N1)	304.5	14.89	12.5	53.37
VA/19(H1N1)	nt	11.26	nt	27.56
duck/MW/18(H1N1)	152	1.82	6	17.44
duck/NJ/78(H2N3)	304.5	21.02	197.5	98.72
duck/PA/84(H5N2)	NN	10.57	NN	NN
swine/HK/98(H9N2)	NN	119.09	NN	NN

C

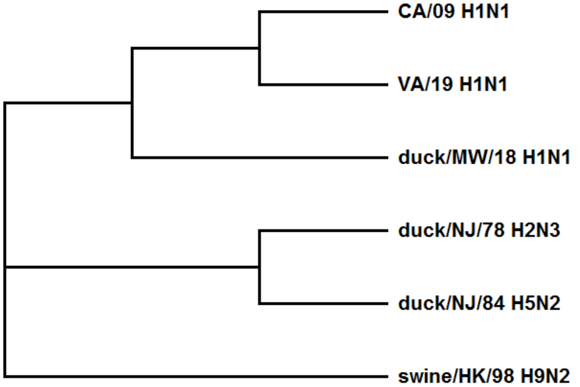

Figure S2: Influenza A viruses used in this study, their phylogenetic analysis and results of microneutralization assay. (**A**) Summary of IAV used for MN and *in vivo* experiments. ma – mouse adapted. (**B**) IC_{50} values of VHH-Fc and their monomeric form. MN analysis was performed on Caco2 cell line in quadruplicate. nt – not tested, NN – non-neutralize. (**C**) Phylogenetic analysis of IAV used for MN in this study. Sequences of the FL HA protein were and aligned using the MEGA X Software (Penn State University, USA) with MUSCLE method. The phylogenetic tree was produced using the maximum likelihood method and visualized in the MEGA X program.

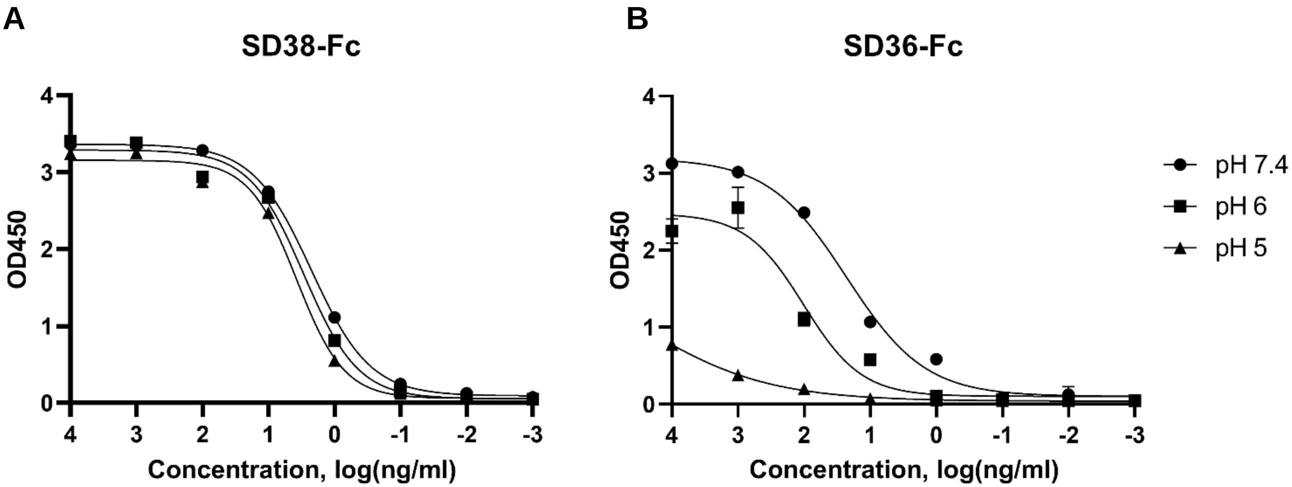

Figure S3: Low pH-induced conformation changes ELISA. ELISA with rFL HA H1 subjected to trypsinization and treated with low pH (citrate buffer pH 7.4, 6 and 5). (**A**) SD38-Fc; (**B**) SD36-Fc.

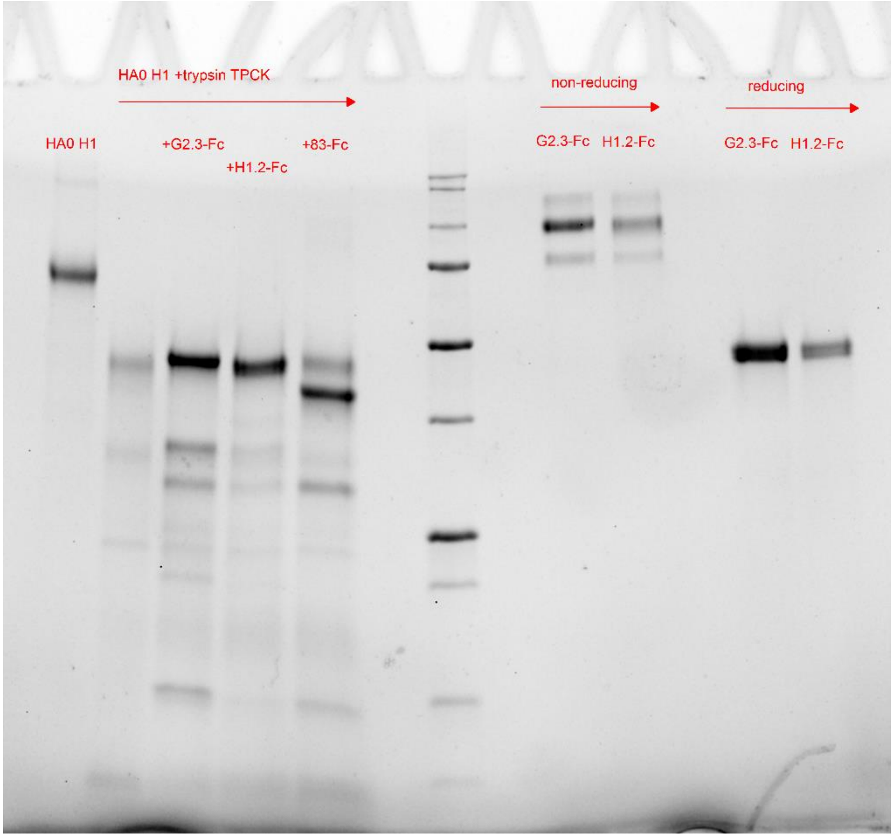

Figure S4: Original image of SDS-PAGE from Figure 1B and Figure 2A.
